# *ERBB4* Confers Risk for Polycystic Ovary Syndrome in Han Chinese

**DOI:** 10.1038/srep42000

**Published:** 2017-02-14

**Authors:** Yingqian Peng, Wei Zhang, Ping Yang, Ye Tian, Shizhen Su, Changming Zhang, Zi-Jiang Chen, Han Zhao

**Affiliations:** 1 Center for Reproductive Medicine, Shandong Provincial Hospital Affiliated to Shandong University, Jinan, China; 2 National Research Center for Assisted Reproductive Technology and Reproductive Genetics, China; 3 The Key laboratory for Reproductive Endocrinology of Ministry of Education, China; 4 Department of joint and bone oncology, Shandong Provincial Hospital Affiliated to Shandong University, Jinan, China; 5 Shanghai Key Laboratory for Assisted Reproduction and Reproductive Genetics, Center for Reproductive Medicine, Renji Hospital, School of Medicine, Shanghai Jiao Tong University, Shanghai, China; 6 Department of Gynecology and Obstetrics, Tianjin Medical University General Hospital, Tianjin, China.

## Abstract

A recent genome-wide association study (GWAS) of polycystic ovary syndrome (PCOS) in European cohorts has identified six susceptibility loci mapping to 11q22.1 (*YAP1*), 2p21 (*THADA*), 11p14.1 (*FSHB*), 2q34 (*ERBB4*), 12q21.2 (*KRR1*), and 5q31.1 (*RAD50*). The loci of 11q22.1, 2p21 and 11p14.1 have been confirmed to be associated with PCOS in Chinese; whereas the other three new loci (2q34, 12q21.2, and 5q31.1) still need to be evaluated in Chinese. This study was aimed to determine if the three new loci identified in European PCOS also confer risks for PCOS in Han Chinese. We performed a case-control genetic association study comprising 1500 PCOS cases and 1220 age-matched control subjects. Marker SNPs rs1351592 (2q34, *ERBB4*), rs1275468 (12q21.2, *KRR1*) and rs13164856 (5q31.1, *RAD50*) were genotyped using TaqMan-MGB probe assay. Genotyping analysis showed the allele frequency of rs1351592 in gene *ERBB4* was significantly different (*P* = 1.05E-03) between PCOS cases and control group, and remained significant even after BMI adjustment (*P*_adjusted_ = 2.09E-04). However, the allele frequencies of the other two risk variants, rs1275468 (12q21.2, *KRR1*) and rs13164856 (5q31.1, *RAD50*), were not significantly different in the replication cohort. Our results demonstrate that *ERBB4*, with the strongest association in European PCOS, also confers risk for PCOS in Han Chinese.

Polycystic ovary syndrome (PCOS) is the most common endocrinopathy with the prevalence of 6–10% in reproductive-aged women^1^. It is a heterogeneous disorder defined by three cardinal clinical features: oligo-ovulation/anovulation (OA), clinical and/or biochemical hyperandrogenism (HA) and polycystic ovaries (PCO)^2^.

Previously in 2011 and 2012, two genome-wide association studies (GWAS) on PCOS in Chinese have identified 11 susceptibility loci mapping to genes functioning in metabolism (*INSR, HMGA2, THADA, RAB5B/SUOX*), follicle maturation and ovulation (*LHCGR, FSHR*) and cell proliferation (*DENND1A, C9orf3, YAP1, TOX3, SUMO1P1*)^3^,^4^ *et al*. Among them, several loci, such as 19p13.3 (*INSR*), 2p21 (*THADA*), 12q13.2 (*RAB5B/SUOX*), 2p16.3 (*LHCGR*), and 9q33.3 (*DENND1A*), have been replicated in European, Egyptian and Indian populations^5^,^6^,^7^. In 2015, Hayes *et al*. have identified two novel loci mapping to 8p32.1 (G*ATA4* and *NEIL2*) and 11p14.1 (*FSHB*) in European ancestry^8^. A subsequent study followed and verified the susceptibility of *FSHB* in PCOS of Han Chinese population^9^.

Recently, Day *et al*. identified six PCOS susceptibility loci that mapped to the genomic areas of 11q22.1 (*YAP1*), 2p21 (*THADA*), 11p14.1 (*FSHB*), 2q34 (*ERBB4*), 5q31.1 (*RAD50*) and 12q21.2 (*KRR1*) in a large cohort of European PCOS^10^. The loci mapped to *YAP1* and *THADA* have been previously reported in Chinese^4^; and the locus of 11p14.1, (*FSHB*) has been identified by Hayes’s GWAS^8^ and replicated in Chinese^9^. But the role of the rest of the three loci (2q34, *ERBB4*; 5q31.1, *RAD50* and 12q21.2, *KRR1*) has been unknown in Chinese.

Replication studies performed in different populations have manifested that there are common genetic risk factors for PCOS across ethnicities^6^,^11^,^12^,^13^,^14^. In this study, we sought to detect if the other three novel identified loci (2q34, rs1351592; 12q21.2, rs1275468 and 5q31.1, rs13164856) confer risks for PCOS in Han Chinese.

## Results

### Basic clinical characteristics

This study consisted of 1500 Han Chinese PCOS women and 1220 age-matched controls [29(27–32) vs 29(25–34), *P* = 0.396]. Basic clinical characteristics of PCOS and control subjects were presented in Table 1. Compared with the controls, group of PCOS subjects had a higher body mass index [BMI, 24.22(21.47–27.73) kg/m^2^ vs 21.88(19.95–24.12) kg/m^2^, *P* < 0.001], a higher testosterone level [T, 43.73(31.49–54.15) ng/dl vs 27.65(19.98–38.18) ng/dl, *P* < 0.001] and a higher luteinizing hormone level [LH, 9.36(6.12–13.39) IU/L vs 4.70(3.51–6.01) IU/L, *P* < 0.001].

### SNP rs1351592 was associated with PCOS in Han Chinese

Allele and genotype frequencies for the analyzed SNPs were depicted in Table 2 and Supplemental Table S1. The observed allele frequencies were in consistent with Hardy–Weinberg equilibrium both in PCOS cases and controls. Among the three SNPs, minor allele frequency (MAF) of rs1351592 in *ERBB4* locus was significantly associated with PCOS with odds ratio (OR) of 1.310 (95%CI: 1.114–1.540, *P* = 1.05E-03). Individuals carrying minor G allele of rs1351592 were at a higher risk to develop PCOS. This variant was strongly correlated in both European and Chinese PCOS populations. Adjustment of BMI by logistic regression analysis did not materially alter this association (*P*_adjusted_ = 2.09E-04). The MAFs of the other two variants, rs1275468 in gene *KRR1* and rs13164856 in gene *RAD50*, did not show any significant differences.

### SNP rs1351592 was associated with PCOS by subtype analysis

All 1500 PCOS fulfilled Rotterdam criteria comprised of four subtypes according to the clinical characteristics: OA + HA (n = 9), OA + PCO (n = 1044), HA + PCO (n = 16), OA + HA + PCO (n = 352). The MAFs of the three SNPs were further analyzed in two subtypes (OA + PCO; OA + HA + PCO); whereas other two subtypes (OA + HA; HA + PCO) were not investigated due to the small sample sizes. Subtype analysis showed that allele frequency of SNP rs1351592 in each subgroup was significantly different from the controls, but not for the other two SNPs, rs1275468 and rs13164856 (Table 3).

### No association between rs1351592 and clinical characteristics

The additive model, dominant model and recessive model were used to compare the distribution of genotype frequencies of rs1351592 between PCOS and control subjects. Of the three models, the dominant model was the most effective for genotype analysis (Supplemental Table S1). Subsequently, clinical characteristics of PCOS cases were further analyzed (Supplemental Table S2) using dominant model (+/+ plus+/− vs. −/−). However, no association was observed among hormonal and metabolic parameters (FSH, LH, T, DHEAS, FG, FINS, and HOMA-IR) between rs1351592 risk allele group and non-risk allele group.

## Discussion

In this study, we evaluated whether the variants rs1351592 (2q34, *ERBB4*), rs1275468 (12q21.2, *KRR1*) and rs13164856 (5q31.1, *RAD50*) recently identified in a European GWAS for PCOS also confer risks for PCOS in Han Chinese. Genotypes of the three SNPs were examined and in view of the current evidence, we verified that the association of the rs1351592 G allele in the gene *ERBB4* had an increased PCOS susceptibility, suggesting that *ERBB4* is likely to play a vital role in the etiology of PCOS across populations.

Our study is the first to replicate the association of rs1351592, rs1275468 and rs13164856 with PCOS in Han Chinese. In regard to our study, a statistically significant association was validated in Chinese by comparing allele frequency of rs1351592 in *ERBB4* locus with the presence of PCOS. This finding suggests shared genetic susceptibility between Han Chinese and European PCOS.

The variant rs1351592 is located within the gene *ERBB4,* which is also known as human epidermal receptors 4 (*HER 4*). *ERBB4* is a member of epidermal growth factor receptors (EGFRs) in human; the other EGFRs involve *ERBB1, ERBB2/HER2*, and *ERBB3/HER3*^15^. Epiregulin (EGFRs ligand) could bind to ERBB1 and ERBB4, and stimulate ERBB2 and ERBB3 by ligand-induced heterodimerization with a cognate receptor. Epiregulin contributes to inflammation, atherosclerosis and oocyte maturation by regulating vascular remodeling and stimulating cell proliferation^16^. EGFR signaling also triggers LH-induced steroidogenesis and then regulated steroid-mediated oocyte maturation. Steroid-mediated maturation may be important in disorders of androgen excess, such as PCOS. Perhaps regulating *EGFR* activity in the ovary might be beneficial for women with androgen excess^17^. Moreover, HER4 signaling may involve in regulating luteal growth^18^.

However, the current data did not replicate the association at the 5q31.1 (rs1275468 in *KRR1*) and the 12p21.2 (rs13164856 in *RAD50*) loci. It is possible that the linkage disequilibrium (LD) structure in the Chinese populations differ from that in the European; so that the significant association had not been detected in the genotyped SNPs.

PCOS is a heterogeneous disorder with a variable clinical picture ranging from reproductive to metabolic dysfunctions. However, the underlying pathologies of diversities in PCOS phenotypes are complicated considering both genetic and non-genetic factors^19^. The role of most European GWAS variants in the etiology of PCOS remains uncertain. Further studies will be needed to determine the role of the variant in the pathogenesis of PCOS. It is hoped that identification and further study of PCOS susceptibility genes will eventually help us better prevent and reduce the consequences associated with the PCOS phenotype, and improve our ability to diagnose and treat this common disorder.

In conclusion, the current replication study confirmed the association of rs1351592 (*ERBB4*) with PCOS in Han Chinese. These data would stimulate interests in exploring more meaningful insights on the role of gene *ERBB4* towards PCOS.

## Materials and Methods

### Subjects

A cohort of 1500 PCOS cases and 1220 age-matched control women of Han Chinese were recruited from the Center for Reproductive Medicine, Shandong University. All subjects diagnosed with PCOS fulfilled the Rotterdam criteria^2^ and thus had at least two of the following three features: OA (anovulation, menstrual cycle >35 d in length), clinical and/or biochemical evidence of HA (hyperandrogenism) and PCO (polycystic ovaries) ultrasound findings (either ≥12 follicles with a diameter of 2–9 mm in at least one ovary or increased ovarian volume >10 ml). Clinical HA was assessed using Ferriman-Gallwey score ≥6. Biochemical HA was defined by elevated total serum concentration of T ≥ 60 ng/dl^3^. Other known endocrinopathies, such as congenital adrenal hyperplasia, androgen-secreting tumors, Cushing’s syndrome, 21-hydroxylase deficiency, thyroid disease and hyperprolactinemia, resulting in the similar presentations, were excluded. Controls were healthy females with regular menstrual cycles, no evidence of HA and PCO.

### Ethical approval

This study was approved by the Institutional Review Board for Reproductive Medicine of Shandong University. All the methods described here were carried out in accordance with the guidelines and regulations approved by the Institutional Review Board of Reproductive Medicine of Shandong University. All participants gave written informed consents.

### Measurements

All subjects underwent a standardized clinical and biochemical measurements. Age and anthropometric measurements, including height and weight, were recorded. Calculation of body mass index (BMI) was according to the formula: weight (kg)/ the squared height (m^2^). Circulating levels of follicle stimulating hormone (FSH), luteinizing hormone (LH) and total testosterone (T) were measured from fasting blood samples at day 2–4 of menstrual cycle. The glucose metabolic and lipid measurements were conducted for PCOS patients. The concentrations of plasma baseline insulin and glucose were detected from a morning fasting blood sample. Insulin resistance was evaluated by the homeostasis model assessment (HOMA-IR) using fasting glucose (FG, mmol/l)*fasting insulin (FINS, mIU/L)/22.5.

### Genotyping

We genotyped three marker SNPs: rs1351592 (2q34, *ERBB4*), rs1275468 (12q21.2, *KRR1*) and rs13164856 (5q31.1, *RAD50*). Genomic DNA was extracted from peripheral whole blood with QIAamp DNA mini kit following the manufacturer’s instructions (QIAGEN, Hilden, Germany). For the replication study, TaqMan-MGB probe assay (Thermo Fisher Scientific, Waltham, USA) was used for genotyping. The assay informations was shown as a Supplementary Table S3. Amplification reactions and genotype detection were performed on 384-well plates with Roche Lightcycle 480. Reaction conditions carried out as follows: pre-incubation at 95 °C for 10 min, followed by 45 cycles of 15 s denaturation at 95 °C, and then annealing, extension and detection at 60 °C for 60 s.

### Statistical analysis

Continuous variables of clinical characteristics between PCOS cases and controls were normally analyzed according to Mann-Whitney U-test and data were presented as median (interquartile range). Hardy–Weinberg equilibrium was evaluated using Haploview software (Broad Institute, Cambridge, MA, USA). Categorical variables were summarized as frequencies. Chi-square test was employed to compare the distribution of allele frequencies between case and control subjects and the results were adjusted by BMI using logistic regression model. OR (odds ratio) and 95%CI (confidence interval) are presented.

The genetic test power of association between the three SNPs and PCOS was calculated by Genetic Power Calculator (http://pngu.mgh.harvard.edu/purcell/gpc/). The sample size of 1500 cases and 1220 controls has enough power (above 90%) to detect association of risk allele frequency of 0.05 with PCOS at relative risk of 1.8.

Genetic models were divided into additive (+/+ vs. +/− vs. −/−), dominant (+/+ plus +/− vs. −/−) and recessive (+/+ vs. +/− plus −/−). Within the subjects with PCOS, Genotype-phenotype correlation was performed using the dominant model. Continuous variables following normal distribution were analyzed by Unpaired Student’s t-tests and continuous variables not normally distributed were compared by Mann-Whitney U-test. These analyses were carried out by SPSS version 23.0 (SPSS Inc., Chicago, IL, USA), a two-tailed value of P < 0.05 was considered statistically significant.

## Additional Information

**How to cite this article:** Peng, Y. *et al. ERBB4* Confers Risk for Polycystic Ovary Syndrome in Han Chinese. *Sci. Rep.* **7**, 42000; doi: 10.1038/srep42000 (2017).

**Publisher's note:** Springer Nature remains neutral with regard to jurisdictional claims in published maps and institutional affiliations.

## Supplementary Material

Supplementary Table

## Figures and Tables

**Table 1:** The clinical characteristics of PCOS patients and control subjects.

	PCOS	Control	*P*
**N**	1500	1220	
**Age**^a^ **(years)**	29(27–32)	29(25–34)	0.396
**BMI**^a^ **(kg/m**^**2**^)	24.22(21.47–27.73)	21.88(19.95–24.12)	<0.001
**FSH**^a^ **(IU/L)**	6.12(5.16–7.08)	6.86(5.92–8.33)	<0.001
**LH**^a^ **(IU/L)**	9.36(6.12–13.39)	4.70(3.51–6.01)	<0.001
**T**^a^ **(ng/dl)**	43.73(31.49–54.15)	27.65(19.98–38.18)	<0.001

BMI: body mass index. FSH: follicle stimulating hormone. LH: luteinizing hormone. T: testosterone.

^a^Data are presented as median (interquartile ranges).

**Table 2:** Allele frequencies in PCOS cases and controls.

SNPs	Allele	PCOS	Control	OR	*P*	*P*_*adj*_
rs1351592	**G**/C	0.143	0.113	1.310(1.114–1.540)	1.05E-03	2.09E-04
rs1275468	**C**/T	0.615	0.598	1.074(0.963–1.198)	0.202	0.095
rs13164856	**T**/C	0.767	0.751	1.088(0.960–1.233)	0.185	0.268

*P*_*adj*_: calculated by logistic regression analysis taking BMI as covariant.

Bold face indicates the effect allele.

**Table 3:** Allele and Genotype analysis in different subgroups of PCOS.

Subgroups	N	Genotype (GG/GC/CC)	MAF	*P*	OR(95%CI)	*P*_*adj*_
Total PCOS	1500	33/362/1105	0.143	1.05E-03	1.310(1.114–1.540)	2.09E-04
OA + HA	9	0/3/6	—	—	—	—
OA + PCO	1044	18/248/778	0.136	1.75E-02	1.239(1.038–1.480)	3.85E-03
HA + PCO	16	0/6/10	—	—	—	—
OA + HA + PCO	352	13/84/255	0.156	1.90E-03	1.458(1.148–1.852)	9.97E-04

*P*_*adj*_: calculated by logistic regression analysis taking BMI as covariant.
