# Supplementary material for: ERBB4 Confers Risk for Polycystic Ovary Syndrome in Han Chinese
Source: Sci Rep. 2017 Feb 14;7:42000. doi: 10.1038/srep42000 (PMC5307312; doi:10.1038/srep42000)
Supplement: Supplementary Table [file srep42000-s1.doc]

**Title Page**

**Title:** *ERBB4* Confers Risk for Polycystic Ovary Syndrome in Han Chinese

**Authors’ names and institutions:**

**Yingqian Peng^1^**^†^**, Wei Zhang^2^**^†^**, Ping Yang^1†^, Ye Tian^1, 3, 4^, Shizhen Su^1^, Changming Zhang^1^, Zi-Jiang Chen****^1, 3^****, Han Zhao^1*^**

^1^ Center for Reproductive Medicine, Shandong Provincial Hospital Affiliated to Shandong University, Jinan, China; National Research Center for Assisted Reproductive Technology and Reproductive Genetics, China; The Key laboratory for Reproductive Endocrinology of Ministry of Education, China.

^2^ Department of joint and bone oncology, Shandong Provincial Hospital Affiliated to Shandong University, Jinan, China.

^3^ Shanghai Key Laboratory for Assisted Reproduction and Reproductive Genetics, Center for Reproductive Medicine, Renji Hospital, School of Medicine, Shanghai Jiao Tong University, Shanghai, China.

^4^ Department of Gynecology and Obstetrics, Tianjin Medical University General Hospital.

**Supplemental table S1.** Genotype frequencies in PCOS cases and Controls.

| **SNP** | **Genotype** | **PCOS** | **Control** | **χ^2^** | ***P_add_*** | ***P_dom_*** | ***P_rec_*** |
| --- | --- | --- | --- | --- | --- | --- | --- |
| **rs1351592** | GG/GC/CC | 33/362/1105 | 17/241/962 | 10.582 | **5.04E-03** | **1.64E-03** | 0.119 |
|  |  | 0.022/0.241/0.737 | 0.014/0.197/0.789 |  |  |  |  |
| **rs1275468** | CC/CT/TT | 554/738/208 | 439/582/199 | 3.164 | 0.206 | 0.075 | 0.609 |
|  |  | 0.369/0.492/0.139 | 0.360/0.477/0.163 |  |  |  |  |
| **rs13164856** | TT/TC/CC | 883/534/83 | 676/481/63 | 4.213 | 0.122 | 0.671 | 0.070 |
|  |  | 0.589/0.356/0.055 | 0.554/0.394/0.052 |  |  |  |  |

P_add_: P value of additive model (three genotypes).

P_dom_: P value of dominant model [(homozygotes of risk allele＋ heterozygotes) vs. Homozygotes of non-risk allele]

P_rec_: P value of recessive model [homozygotes of risk allele vs. (Heterozygotes ＋ Homozygotes of non-risk allele)]

**Supplemental table S2.** Comparison of characteristics in PCOS cases using dominant model of rs1351592.

| **Characteristics** | **Risk allele**  **group (N=395)** | **Non risk-**  **allele group(N=1105)** | ***P*** |
| --- | --- | --- | --- |
| **Age** ^a^ **(years)** | 29(27-32) | 30(27-32) | 0.181 |
| **BMI** ^a^ **(kg/m^2^)** | 23.88(21.28-27.34) | 24.31(21.48-27.93) | 0.176 |
| **FSH** ^b^ **(IU/L)** | 6.04±1.63 | 6.12±1.63 | 0.474 |
| **LH** ^a^ **(IU/L)** | 9.46(6.15-13.13) | 9.28(6.07-13.51) | 0.938 |
| **T** ^a^ **(ng/dl)** | 44.40(32.56-54.79) | 43.64(31.07-53.78) | 0.427 |
| **Glu0’** ^a^ **(mmol/l)** | 5.39(5.10-5.77) | 5.42(5.14-5.75) | 0.447 |
| **Glu120’** ^a^ **(mmol/l)** | 6.22(5.31-7.32) | 6.31(5.40-7.40) | 0.410 |
| **INS0’** ^a^ **(mIU/L)** | 11.48(8.13-16.54) | 11.87(8.06-17.36) | 0.675 |
| **INS120’** ^a^ **(mIU/L)** | 51.97(30.64-92.39) | 52.28(30.63-92.68) | 0.857 |
| **HOMA-IR** ^a^ | 2.80(1.88-4.10) | 2.88(1.92-4.31) | 0.535 |

^a^ Data are presented as median (interquartile ranges) for continuous variables not normally distributed.

^b^ Data are presented as mean ± SD for continuous variables following normal distribution.

**Supplemental table S3.** The Assay Informations of the three SNPs.

| **SNPs** | **Assay ID** | **Catalog #** |
| --- | --- | --- |
| **rs1351592** | C___8836282_10 | 4351376 |
| **rs1275468** | C___8711805_10 | 4351376 |
| **rs13164856** | C__11818625_10 | 4351376 |
